# Assessment and Prediction of Water Resources Vulnerability Based on a NRS-RF Model: A Case Study of the Song-Liao River Basin, China

**DOI:** 10.3390/e23070882

**Published:** 2021-07-11

**Authors:** Weizhong Chen, Yan Chen, Yazhong Feng

**Affiliations:** 1 College of Economics and Management, Nanjing Forestry University, Nanjing 210037, China; chenweizhong0713@163.com (W.C.); fengyazhong@njfu.edu.cn (Y.F.); 2 Academy of Chinese Ecological Progress and Forestry Development Studies, Nanjing Forestry University, Nanjing 210037, China

**Keywords:** basin water resources, vulnerability assessment, neighborhood rough set, random forest regression, scenario prediction

## Abstract

The vulnerability of water resources is an important criterion for evaluating the carrying capacity of water resources systems under the influence of climate change and human activities. Moreover, assessment and prediction of river basins’ water resources vulnerability are important means to assess the water resources security state of river basins and identify possible problems in future water resources systems. Based on the constructed indicator system of water resources vulnerability assessment in Song-Liao River Basin, this paper uses the neighborhood rough set (abbreviated as NRS) method to reduce the dimensionality of the original indicator system to remove redundant attributes. Then, assessment indicators’ standard values after dimensionality reduction are taken as the evaluation sample, and the random forest regression (abbreviated as RF) model is used to assess the water resources vulnerability of the river basin. Finally, based on data under three different future climate and socio-economic scenarios, scenario predictions are made on the vulnerability of future water resources. The results show that the overall water resources vulnerability of the Song-Liao River Basin has not improved significantly in the past 18 years, and the overall vulnerability of the Song-Liao River Basin is in the level V of moderate to high vulnerability. In the future scenario 1, the overall water resources vulnerability of the river basin will improve, and it is expected to achieve an improvement to the level III of moderate to low vulnerability. At the same time, the natural vulnerability and vulnerability of carrying capacity will increase significantly in the future, and the man-made vulnerability will increase slowly, which will deteriorate to the level V of moderate to high vulnerability under Scenario 3. Therefore, taking active measures can significantly reduce the vulnerability of nature and carrying capacity, but man-made vulnerability will become a bottleneck restricting the fragility of the overall water resources of the river basin in the future.

## 1. Introduction

As an indispensable natural resource for human survival and social development, water resources play an extremely important role in the sustainable development of a viable socio-economy. However, water is also a limited resource that is unevenly distributed in time and space. Under the influence of increasing human activities and rapid urban development, water environmental pollution and water shortages have always plagued human beings and have made the sustainable development of water resources in river basins appear vulnerable [1]. As one of China’s important water systems, although the Song-Liao River Basin has relatively richer water resources than Northwest and North China, there are problems such as uneven temporal and spatial distribution and prominent contradictions between water supply and demand. Also, the distribution of precipitation in this basin is extremely uneven [2]. Furthermore, water resource utilization has low efficiency and serious waste. At present, rivers in this basin have poor self-purification ability [3]. Also, water pollution has become an important factor that restricts the economic and social development of Song-Liao River Basin [4]. Many of the above factors have contributed to the increase in the vulnerability of water resources in the basin. Therefore, it is necessary to conduct in-depth research on the vulnerability of water resources in the Song-Liao River Basin in order to better manage the water resources to reduce the vulnerability.

The concept of water resources vulnerability originated from the related studies on groundwater vulnerability proposed by the French scholar Margat [5]. After the enrichment and development of Xia et al. [6,7,8], the connotation and scope of water resources vulnerability research continue to expand, and a rich research system on the vulnerability of water resources systems has been formed. In terms of research object selection, not all scholars chose general water resources system as the research object. Many scholars studied the vulnerability of various specific components of water resources systems, such as surface water [9,10], ground water [11,12], drinking water [13], household water [14], urban runoff [15] and water quantity [16]. What’s more, there are also many scholars who have combined water resources with social, economic, ecological and other external factors to conduct research on vulnerability [17,18]. In terms of research perspective selection, many scholars currently choose to study the vulnerability of water resources from the perspective of climate and land, that is, to study the impact of climate change and land use on water resources vulnerability [19,20,21]. In terms of research frameworks, some frameworks such as the 2014 Intergovernmental Panel for Climate Change (IPCC) framework is applied to the research on vulnerability in the context of climate change [22]. Combining existing relevant research literature [23,24,25], the authors believe that water resources vulnerability is the degree to which the water resources system is susceptible to and unable to cope with the adverse effects of the external natural climate change and human socio-economic activities. In this paper, the water resources system mentioned in the definition of water resources vulnerability refers to various water bodies (such as rivers, lakes, reservoirs, groundwater, etc.) and related engineering buildings that are related to each other in hydrology, hydraulics, and water conservancy within a basin or region.

The current research on water resources vulnerability includes quantitative research and qualitative research. There are few qualitative studies, which mainly focus on the theoretical framework and tools related to water resources vulnerability [22,26]. At present, most of the research related to the vulnerability of water resources focuses on the quantitative model research, which mainly includes the evaluation and prediction of the vulnerability of the water resources system. In terms of evaluation, the functional evaluation method and indicator evaluation method are used a lot. Among them, the function evaluation method [27] has a clear operation mechanism and is easy to evaluate multiple regions, but there are problems such as difficulty in interpreting the results. Therefore, at present, scholars have published relatively few studies on this method. Compared with the function method, the index evaluation method is flexible, easy to explain, clear in structure, and compatible with multiple model methods, so most of the current studies are carried out on the basis of indicator evaluations. Regarding the selection of water resources vulnerability indicators, there is no unified objective standard. Scholars have combined different evaluation objects and theoretical connotations to construct corresponding indicators, and they used a variety of methods to evaluate water resources vulnerability. In the application of evaluation methods, weight evaluation methods, such as entropy weight method [28], projection pursuit method [29], analytic hierarchy process (AHP) method [30] are used more frequently. In addition, principal component analysis (PCA) [31,32], technique for order preference by similarity to an ideal solution (TOPSIS) [33], rough set cloud [34], connection entropy approach [35], expert-based model [36] and other quantitative methods are also used in the assessment of water resources vulnerability. What’s more, Geographical Information System (GIS) is often used in the study of water resources vulnerability [37]. Many scholars have improved the GIS model and combined it with AHP [37,38] and F-Hydra [39]. Among them, GIS and the integrated climatic modelling framework (geographical spatially distributed water balance model (GEO-CWB)) model developed and verified by Trinity College (Dublin, Ireland) are used together in the study of water resources vulnerability [40]. Furthermore, some indicator models are used for the vulnerability assessment and mapping. Among them, the indicator models such as DRASTIC (depth to groundwater, recharge rate, aquifer, soil, topography, vadose zone’s impact, aquifer’s hydraulic conductivity) model [41,42,43,44], SINTACS (Soggiacence, actual infiltration, non-saturated zone, typology of overburden, hydrogeological characteristics of the aquifer, hydraulic conductivity, topographic Slope) model [45], GALDIT model (an abbreviation for the six parameters of groundwater occurrence, aquifer hydraulic conductivity, level of groundwater above sea level, distance from the shoreline, impact of the existing status of seawater intrusion, and thickness of the aquifer) [46] and the susceptibility index (SI) method [47] are the most used ones. There are also some methods for evaluating the vulnerability of karst water, such as epikarst, protective cover, infiltration conditions, karst network development (EPIK), protective cover, infiltration conditions (PI), concentration of flow, overlaying layers, precipitation (COP) and altitude, slope, lithology, infiltration, soil (APLIS) methods [48,49]. For the prediction of water resources vulnerability, the number of evaluation models is also considerable. In addition to the indicator method and function method related to water resources vulnerability evaluation used in the prediction, there are other efficient prediction methods and technologies, such as the Monte Carlo model [50], soil and water assessment tool-water evaluation and planning system (SWAT-WEAP) coupling method [51], Arc-Wofe system simulation method [52], enriching the existing prediction method system of water resources vulnerability.

At present, the research on the vulnerability of water resources in river basins has achieved fruitful results. The connotation of water resources has been continuously enriched and developed, and the corresponding evaluation methods have become increasingly complete. Specifically, the evaluation content has realized the transformation from a single water quality study to a comprehensive system evaluation combining natural climate, ecological, and socio-economic systems. In the past, the indicator evaluation and prediction of water resources vulnerability had the following problems. First, in terms of the construction of the indicator system, some researched indicator settings were insufficiently targeted. What’s more, there are many and complex indicators involved in constructing a water resources vulnerability indicator system, and the interrelationships between indicators are closely related. Therefore, problems such as multi-index data redundancy, inter-index correlation and duplicate information are easy to appear. If they are not handled properly, these problems will cause a certain interference to the accuracy of the evaluation results objectively. Second, most of the vulnerability research of water resources is in terms of evaluation and relatively little research is in terms of prediction. As a result, management control has become a post-control, and it is difficult to make timely preparations for adjustment and control in advance. Third, for the data to be predicted in the future scenario, the accuracy of the model is a problem worthy of attention. In order to more accurately predict the vulnerability of water resources under future scenarios, the comparison and selection of models is crucial.

The Song-Liao River Basin is a densely populated area, a food production base and an important heavy industry base. The vulnerability of water resources in the Song-Liao River Basin deeply affects people’s lives, food and energy production in the basin. In addition, compared with the other five major rivers in China, the rivers in the Song-Liao Basin in the Northeast China have the following unique characteristics: a long dry season, many tributaries, small water flow and long-term freezing on the water surface. These characteristics make the Songhua River and Liao Rivers have poor self-purification ability and be more susceptible to pollution. However, there are few studies on the overall vulnerability of water resources in it. The existing research focuses on the vulnerability of groundwater resources in the Songhua and Liao River Basin, which mainly uses the indicator evaluation method of the DRASTIC model [53,54]. Other studies on the vulnerability of water resources in the Song-Liao River Basin include the risk assessment of storm flood disasters in the mainstream of the Songhua River [55], the study on the vulnerability of the Songhua River Basin landscape pattern and its driving forces [56], risk assessment of groundwater pollution caused by agricultural activities in the Lower Liao Plain [57] and so on.

In order to better analyze the vulnerability of water resources to better manage the water resources in the Song-Liao River basin, we will do the following tasks: combining the actual conditions of the Song-Liao River Basin, this paper constructs an indicator system for water resources vulnerability assessment in the Song-Liao River Basin. In order to solve the problems of data redundancy and duplicate information in the index system, this paper uses the neighborhood rough set attribute reduction method to reduce the dimensionality of the original index system and remove redundant indicators. Then, this paper uses the standard value of the original indicator level threshold as the input vector and the vulnerability level value as the output vector to construct a random forest model. The assessment indicator system after dimensionality reduction is used as the basis of the random forest model to evaluate and predict the water resources vulnerability of the Song-Liao River Basin. The indicator data under different scenarios in 2025 and 2030 is substituted into the random forest model as input data to obtain the predicted value of water resources vulnerability in 2025 and 2030, so as to provide a reference for future water resources planning and adaptive management.

## 2. Data and Methodology

### 2.1. Research Area

**Figure 1:**
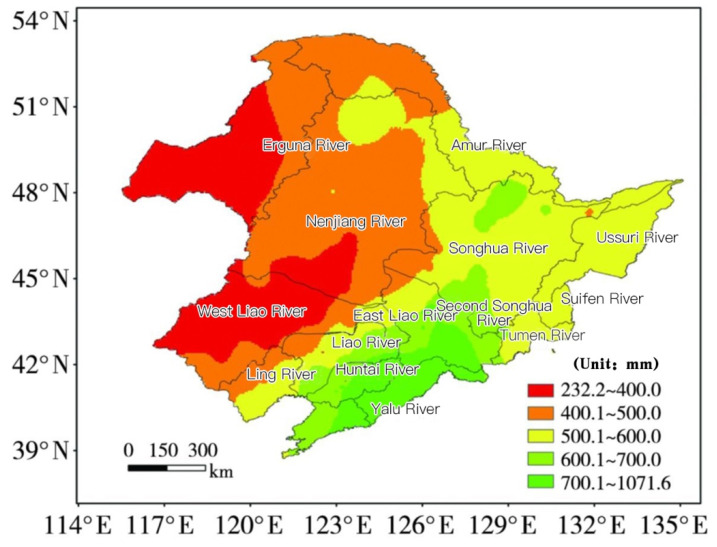
Research area.

The Song-Liao River Basin includes two sub-basins, the Liao River Basin and the Songhua River Basin, whose total area is 1,248,948 km^2^. The basin is surrounded by mountains to the west, north and east, and there is a vast plain in the middle of the basin. The specific distribution of rivers is shown in Figure 1. In the Song-Liao River Basin, that is rich in black soil resources, the plains are flat, the soil is fertile, the rainfall is abundant, the climate is suitable, the sunlight is sufficient, and it has good conditions for agricultural development [58]. What’s more, the basin is also a major industrial hub for coal, steel, petrochemical and equipment manufacturing [59,60,61]. In terms of precipitation, the annual average precipitation in the Song-Liao River Basin shows a spatial distribution characteristic of less precipitation in the northwest and more precipitation in the southeast [2]. The spatial distribution of annual average precipitation in Song-Liao Basin is shown in Figure 1. At present, the Song-Liao River Basin is in a stage of extreme precipitation, and the probability of extreme precipitation has increased [62]. What’s more, due to uneven seasonal precipitation and the high-water level of the drainage channel, the internal water cannot be discharged. The mountainous area has serious soil erosion, and a large amount of sediment is discharged and deposited on the drainage channel, which causes that the riverbed has been silted up year by year, the channel is blocked, and the water is urgent in the flood season. Also, poor water leakage in the waterlogged area and low infiltration capacity have led to frequent occurrence of waterlogging in the watershed. As the northwestern part of the basin is in the westerly zone, when a large precipitation front and low-pressure system pass through this area, the rainfall will be significantly reduced or no precipitation. The normal annual precipitation is below 400 mm, and the water surface evaporation capacity is 1100–1200 mm, which caused many droughts [2]. With the development of industrialization and urbanization and the massive discharge of industrial wastewater and domestic sewage, the Song-Liao River Basin is facing a severe situation of aggravated pollution. According to relevant statistical data such as environmental yearbook, since 2014, the chemical oxygen demand (COD) emissions of the Song-Liao River Basin are about 830,400 tons, and the ammonia and nitrogen emissions are about 12.85 tons. Compared with the previous level, the water pollution problem still cannot be ignored. This objectively aggravates the vulnerability of water resources, so research on the vulnerability of water resources is timely.

### 2.2. Data Sources

In the empirical research part of this article, the water resource vulnerability assessment period is 2000–2017. In the scenario prediction stage, we mainly study the vulnerability of water resources in 2025 and 2030. Also, the data format of all indicators is time series. Among them, some data on indicators related to water resources vulnerability of the Song-Liao River Basin from 2000 to 2017 is derived from the “Song-Liao River Basin Water Resources Bulletin” (2000–2017), “China Statistical Yearbook” (2000–2018), and “China Environmental Statistical Yearbook” (2000–2018) and “China Water Conservancy Yearbook” (2000–2018). What’s more, some indicators need to be calculated by the data directly obtained. For example, the reclamation index is obtained by dividing the cultivated land area by the total land area.

### 2.3. Methodology

In this paper, we construct an indicator system for water resources vulnerability assessment in the Song-Liao River Basin. Then, we use the neighborhood rough set method to reduce the dimensionality of the original index system and remove redundant indicators. Finally, we construct a random forest model to evaluate and predict the water resources vulnerability.

#### 2.3.1. Assessment Indicator System

Water resources refer to available resources or the water source that may be used, which should have sufficient quantity and suitable quality and meet the specific needs of a certain place in a period of time. What’s more, the drainage basin refers to the entire area through which the mainstream and tributary of the river flow. Compared with static areas, its coverage is wider. Therefore, the composition of the vulnerability of water resources in a river basin is more complex, which includes the vulnerability generated in the degradation of the water resources system itself and the recycling process, as well as the vulnerability generated in the development and utilization of water resources. In this paper, starting from the characteristics, manifestations and causes of water vulnerability, combining the situation of the Song-Liao River Basin itself, we construct an assessment indicator system on the framework of nature, man-made and carrying capacity. Therefore, we construct the first-level indicators from the three aspects of natural vulnerability, man-made vulnerability and vulnerability of carrying capacity. In particular, the indicators of natural vulnerability mainly characterize the impact of natural conditions on the quantity and quality of water resources. The indicators of man-made vulnerability mainly characterize the impact of human activities on the quantity and quality of water resources. The indicators of the vulnerability of carrying capacity mainly characterize the carrying capacity of water resources for human social and economic development from the perspective of the demand for water by the human socio-economic system. Then, we construct third-level indicators under each of second-level indicators of water quantity, water quality and disasters. According to the construction principles of the indicator system and the availability and completeness of data, 24 assessment indicators are selected to construct an assessment indicator system for water resources vulnerability finally, which is shown in Table 1.

There is currently no unified classification of the criteria for evaluating the vulnerability of water resources in river basins. Combining the situation of the Song-Liao River Basin and other river basins with similar environmental conditions, such as the Huai River Basin and Hai River Basin, a scale and standard suitable for evaluating the vulnerability of the Song-Liao River Basin has been established by referring to previous studies [3,15,63,64,65] and the current domestic and foreign water resources management standards, such as the evaluation standards of water scarcity established by organizations such as the World Meteorological Organization, the World Food and Agriculture Organization, the United Nations Educational, Scientific, Cultural and Health Organization, and the United Nations Commission for Sustainable Development. This paper divides the vulnerability of water resources into seven levels, ranging from the level I of no vulnerability to the level VII of extreme vulnerability. The specific grade standard is shown in Table 2.

#### 2.3.2. Attribute Reduction Method

The rough set concept was originally proposed by Polish scientist Pawlak [66] in 1982 to deal with data duplication and redundancy. It is a mathematical tool for processing all kinds of data by solving the upper and lower approximate relations. The attribute reduction of a rough set refers to the identification of key information and the removal of redundant information by specific calculation methods without changing the existing decision-making attributes.

The traditional rough set needs to use the discretization method to divide the data before the attribute reduction, so as to achieve the discretization state. However, there are many discretization methods at present, and there is no recognized best processing method for discretization. At the same time, improper processing or external interference in the process of discretization of data may cause data distortion and change the nature of the original data, thereby bringing wrong judgments to decision makers. In order to make up for the shortcomings of the classic rough set, Hu [67] introduced the neighborhood model into rough set to form the neighborhood rough set theory. Neighborhood rough set is proposed to overcome the problem that traditional rough set reduction methods rely on discretized data. Therefore, the method of neighborhood rough set is used to reduce the dimensionality of the original index system in this paper. The related definitions are as follows [68]:

**Definition** **1** *(Measurement)**.** Given the universe*$U=\left\{x_{1},x_{2},\cdots ,x_{n}\right\}$*, if for any*$x_{i},x_{j},x_{k}\in U$*, there are unique real functions ∆ corresponding to them, and ∆ satisfies:*

*1.*  *Non-negative.*$\Delta \left(x_{i},x_{j}\right)\geq 0$*, if and only if*$x_{i}=x_{j}$*,*$\Delta \left(x_{i},x_{j}\right)=0$.
*2.*  *Symmetry.*$\Delta \left(x_{i},x_{j}\right)=\Delta \left(x_{j,}x_{i}\right)$.
*3.*  *Triangle inequality.* $\Delta \left(x_{i},x_{k}\right)\leq \Delta \left(x_{i},x_{j}\right)+\Delta \left(x_{j},x_{k}\right)$.

*∆is called the metric or distance function, and*<U,Δ> is called the metric space.

**Definition** **2** *(Neighborhood)**.** For any metric space* <U,Δ> *and any* U *, the neighborhood* δ *of any* $x_{i}$ *can be expressed as:*

(1) $$\delta \left(x_{i}\right)=\left\{x|x\in U,\Delta \left(x_{i},x_{j}\right)\leq \delta \right\},\delta \geq 0$$

Here the formula of the neighborhood radius is $\delta \left(x_{i}\right)=Std\left(a_{i}\right)/\lambda$; $Std\left(a_{i}\right)$ is the standard deviation of the indicator attribute $a_{i}$; λ is a characteristic parameter, which is set according to the difference in the accuracy of the final reduction. By adjusting and setting the size of λ, the size of the neighborhood $\delta \left(x_{i}\right)$ of each indicator attribute can be changed, and the value is generally [2,4].

**Definition** **3** *(Upper and lower approximation)**.** In the neighborhood rough set decision-making system*$NDS=\left(U,A\cup^{\;}D\right)$*, given the universe*$U=\left\{x_{1},x_{2},\ldots ,x_{n}\right\}$*and the neighborhood relationship*N*on it, that is, two-tuple*NS=(U , N)*, for any* X⊆U*, the lower approximation of*X*in the neighborhood approximation space*NS=(U , N)*is:*

(2) $$NX=\text{\{}x_{i}|\;\delta \left(x_{i}\right)X\;,x_{i}\in U\}$$

At the same time, the lower approximation $\underline{NX}$ of X is also defined as the positive domain, denoted as Pos(X)=NX.

**Definition** **4** *(Neighborhood dependence)**.** In the decision system*$S=\left(U,C\cup^{\;}D\right)$*,*c∈C*, the importance of conditional attribute (indicator)*c*is defined as:*$Sig\left(c\right)=\gamma_{C}\left(D\right)-\gamma_{C-\left\{c\right\}}\left(D\right)$*, where the calculation formula of the dependency*$\gamma_{C}$*of the conditional attribute set*C*relative to the decision attribute*D*is:*

(3) $$\gamma_{C}\left(D\right)=\frac{CARD\left(POS_{C}D\right)}{CARD\left(U\right)}$$

**Definition** **5** *(Knowledge reduction)**.** Given a knowledge base* S=(U,R) *and a set of equivalent relations* P⊆R *on it, for any* G⊆P *, if G satisfies the following conditions:*

*(1)*  G*is independent, that is, every element in*G is indispensable.
*(2)*  IND(G)=IND(P)*, so that the positive domains of*G and P *relative to the decision attribute are the same, that is,* $pos_{G}D=pos_{P}D$*, then* G *is a reduction of* P*, denoted as G ∈ Red (P).*

At present, scholars have gradually increased their research on the attribute reduction algorithm of neighborhood rough set. The attribute reduction algorithm has been optimized to form a rich system, and it has also been extended to the integration with other machine learning methods such as Bayesian networks, neural networks, and support vector machines. The attribute reduction mechanism of the neighborhood rough set is similar to the traditional rough set attribute reduction method. The commonly used method is the attribute reduction method based on importance, which is calculated essentially based on the positive domain of the decision attribute relative to the conditional attribute (that is, the lower approximation). In the process of attribute reduction, if the positive range of an indicator in the assessment indicator system is higher, the removal of the indicator from the constructed assessment indicator system will have a greater impact on the entire assessment system, and the indicator will be given a higher degree of importance. What’s more, the limit of the importance calculated in the attribute reduction of the assessment indicator is 0. In fact, the importance of 0 does not exist, because the constructed indicators have their specific theoretical basis and will have some impact on the overall indicator system to a certain extent. Therefore, choosing a suitable attribute reduction method is very important for the overall assessment of the indicator.

In this paper, the forward greedy algorithm of neighborhood rough set is used for attribute reduction. It retains the indicator with the largest attribute importance and starts to select backwards. Also, it ensures that the core is not reduced. The main algorithm steps are as follows:

*Step 1*: Generate decision tables and determine the values of various model parameters, according to the data set decision system $NDS=\left(U,A\cup^{\;}D\right)$, including discretized decision attributes.
*Step 2*: Calculate and search the neighborhood radius. The optimal neighborhood radius of the conditional attributes of each subsystem is determined respectively, and the neighborhood set of the sample is obtained separately according to the definition of the neighborhood radius. The size of the neighborhood radius is calculated based on the standard deviation of the attribute samples and the relative number of neighborhood parameters. The samples under the same neighborhood radius as sought are regarded as the same-attribute neighborhood set.
*Step 3*: Calculate the upper and lower approximate set, that is, calculate the upper and lower approximations of the decision attribute set relative to the condition attribute set. The lower approximation set is also the positive domain of the neighborhood rough set.
*Step 4*: Calculate the dependency of the decision attribute in each condition attribute subset by the positive domain and calculate the importance of each decision attribute relative to each condition attribute according to the importance solving formula.
*Step 5*: Get the reduction set. The samples whose attribute importance degree exceeds the set appropriate lower limit of importance degree are taken as the final attribute reduction set, so as to obtain a satisfactory solution.

#### 2.3.3. Random Forest Models

Random forest is a relatively novel machine learning model, which has certain applications in many fields [69,70]. The algorithm is a classification regression model composed of multiple decision trees proposed by Breiman [71]. It is constructed by a certain sampling method such as bootstrap sampling method, based on the classification decision tree without pruning constructed by the categorical and regression trees (CART) algorithm, which has higher model accuracy and adaptability compared with the decision tree. The final output of random forest regression uses the average of the output results of a single tree (for the regression model) or the classification result with the most votes obtained by the “simple majority voting method” (for the classification model).

The randomness of random forest is mainly reflected in the randomness of sample extraction and variable extraction. In terms of the randomness of sample extraction, the random forest model randomly selects a certain number of samples from the samples to be trained. Then, it takes the extracted sample as the root node of the tree and construct a corresponding number of decision tree models based on this, and then model training is conducted on this basis. The randomness of variable extraction refers to using the random sampling method to select variables to be extracted that will be used as the split node of the tree when each decision tree model is constructed. When the random forest model is used for data regression prediction, it is necessary to construct a regression model and obtain the output result of a single tree by averaging. Specific steps are as follows:

*Step 1*: Randomly select a decision tree with a number of *k*. The bootstrap method is used to resample the original samples, thereby randomly generating *k* training sets $\theta_{1},\theta_{2},\theta_{3}\ldots \theta_{k}$, that is, the number of trees generated is *k* (that is, the value of parameter *ntree*). At the same time, each training set trained is used to generate the corresponding decision tree $\left\{Tr\left(x,\theta_{1}\right),Tr\left(x,\theta_{2}\right),Tr\left(x,\theta_{3}\right),\ldots ,Tr\left(x,\theta_{k}\right)\right\}$.
*Step 2*: Randomly extract the dimensional feature set with the number *m*, that is, randomly extract *m* features from the dimensional features with the indicator feature number *M* as the split feature set of the current node (that is, the value of parameter *mtry*), and use the standardized mean-square error as the standard to judge whether these *m* features follow the most appropriate split method to carry out splitting, so that the whole after splitting has the best stability.
*Step 3*: Calculate the observation value of a single tree. The prediction of a single decision tree is obtained by the weighted average of the dependent variables’ observed values $Y_{i}\left(i=1,\;2,\;3,\ldots n\right)$.
*Step 4*: Calculate the predicted value of the random forest. According to the weight of each decision tree $\omega_{i}\left(x,\theta_{t}\right)\left(t=1,\;2,\;3\ldots k\right)$, the mean value of the observation value of each decision tree is taken as the final result.

#### 2.3.4. Integration of Neighborhood Rough Set and Random Forest Algorithm

Aiming at the solving the problems of multi-index data redundancy, inter-index correlation and duplicate information in the water resources vulnerability assessment system constructed in this paper, this paper proposes to use the neighborhood rough set method to reduce the dimensionality of the original indicator system to remove redundant attributes. For methods that can remove redundant attributes, there are some methods that can remove redundant attributes such as principal component analysis [31,32] and projection pursuit [29]. Although the use of these methods helps to achieve the dimensionality reduction effect of the indicators, they are not good for the subsequent prediction work. The reasons are as follows. These methods synthesize new indicator values with quantitative methods by combining certain indicators. Also, they do not consider the decision attributes and do not use conditional attributes to judge decision attributes, which may neglect the important information, further reduce the space [72,73]. Therefore, they are difficult to interpret the newly synthesized indicators and to integrate the data in different situations in the future. Also, it will also increase the workload of calculation, so it is not suitable for the study. The neighborhood rough set can effectively make up for these shortcomings [73]. Moreover, the random forest regression algorithm proposed in this paper has further advantages over multiple linear regression, support vector machine regression, neural network, decision tree and other methods, which can effectively improve the accuracy of model prediction and the effect of model construction. The following comparison of these machine learning methods in Section 3.3.2 also confirms this point well.

Therefore, this paper integrates neighborhood rough set and random forest model for the assessment and prediction of water resources vulnerability. Using the neighborhood rough set theory to reduce the dimensionality of the original assessment indicator system can objectively improve the efficiency of the random forest algorithm and the speed of model establishment, which is the basis for the training of the random forest regression model. What’s more, the integration of the two methods can reduce the interference caused by unfavorable assessment indicators that may appear in the research of the paper, thereby improving the effect of the assessment and prediction of the water resources vulnerability.

**Figure 2:**
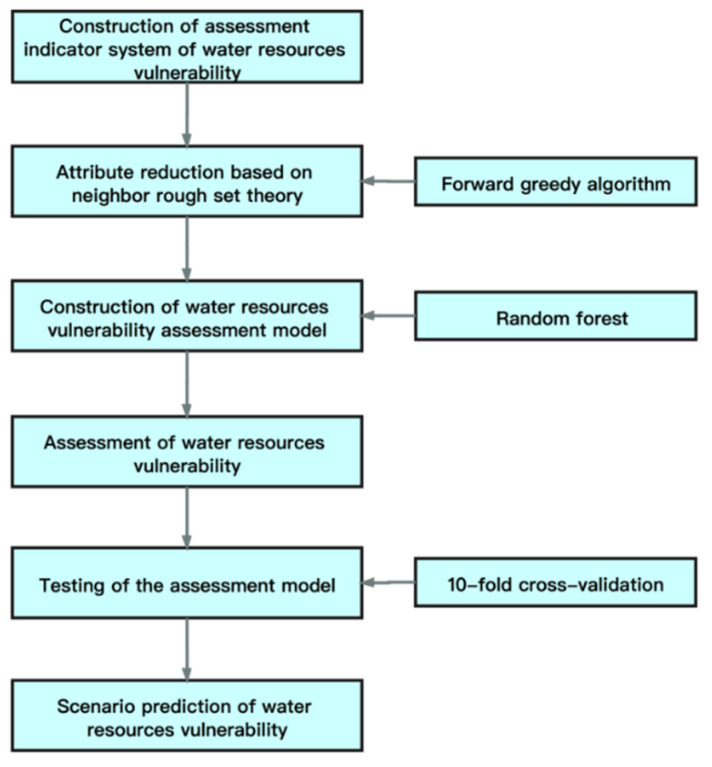
Water resources vulnerability assessment model based on the integration of neighborhood rough set and random forest model.

The integration process of neighborhood rough set and random forest model is shown in Figure 2 and specific steps are as follows:

*Step 1*: Attribute reduction. After constructing the assessment indicator system of water resources vulnerability, we use the forward greedy algorithm of neighborhood rough set to reduce the dimensionality of the original indicator system to remove redundant attributes. It retains the indicators with the greatest attribute importance, and then starts to select it backwards, while also ensuring that the core is not reduced.
*Step 2*: Construction of water resources vulnerability assessment model. We take the standard value of the indicator level threshold of the assessment indicator system after dimensionality reduction as the input vector and the vulnerability level value as the output vector to construct a random forest model.
*Step 3*: Assessment of water resources vulnerability. The indicator data of the Song-Liao River Basin from 2000 to 2017 is substituted into the model to obtain the water resources vulnerability evaluation value in the past few years.
*Step 4*: Testing of the assessment model. We use the 10-fold cross-validation method to test the trained model to judge the reliability of the results. In order to verify whether the accuracy of the random forest regression model is better than other models in this paper, the neural network model, decision tree and support vector machine regression model with excellent nonlinear regression function are compared with it.
*Step 5*: Scenario prediction of water resources vulnerability. The indicator data under different scenarios in 2025 and 2030 is substituted into the random forest model as input data to obtain the predicted value of water resources vulnerability, so as to provide reference for future water resources planning and adaptive management.

## 3. Results and Discussion

### 3.1. Attribute Reduction of Evaluation Indicators

#### 3.1.1. Correlation Analysis of Evaluation Indicators

**Figure 3:**
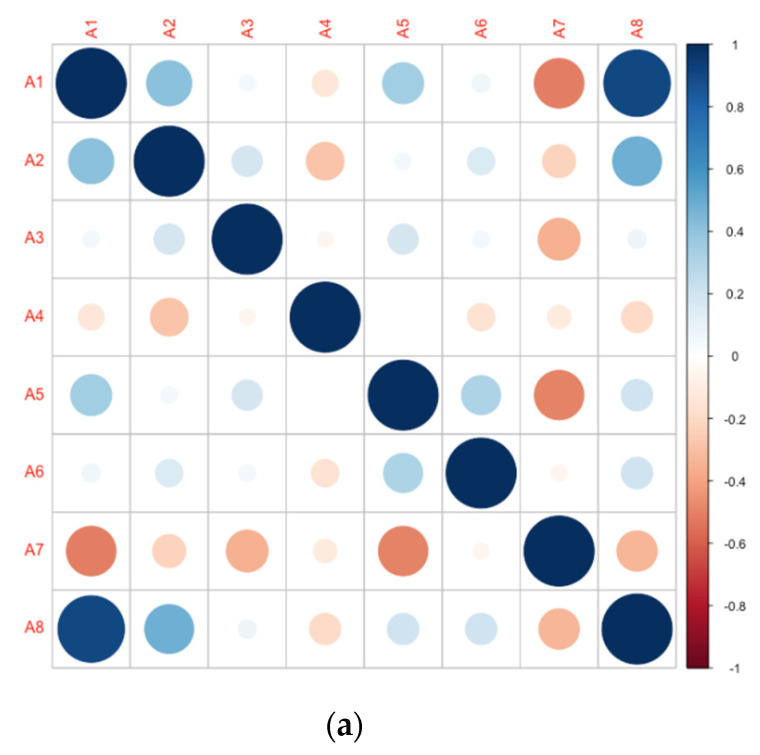

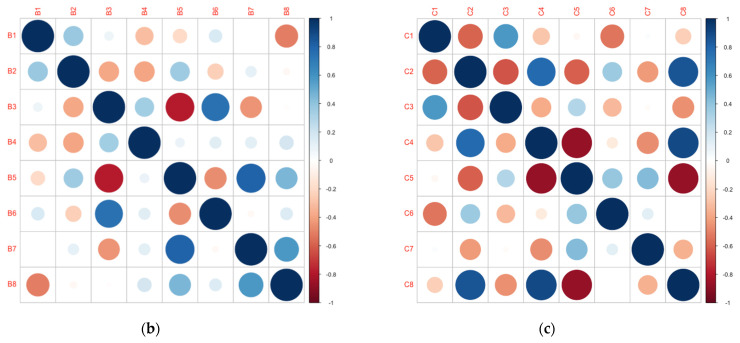
Correlation coefficient diagram of the original index system: (**a**) correlation coefficient diagram of natural vulnerability; (**b**) correlation coefficient diagram of man-made vulnerability; (**c**) correlation coefficient diagram of vulnerability of carrying capacity.

From the above process of establishing the water resources vulnerability assessment indicator system, it can be seen that there are many factors affecting the vulnerability of water resources, and some indicators have greater redundancy and repetition. In order to facilitate the analysis of the repeatability of the indicators, this paper analyzes the correlation between the indicators under each first-level indicator. Each indicator includes 18 years of data in the Song-Liao River Basin from 2000 to 2017. See Figure 3 for the analysis results. Specifically, the correlation coefficient diagram of natural vulnerability is shown in Figure 3a. Most absolute values of the correlation coefficient between indicators are not higher than 0.5. The correlation coefficient between *A*_1_ and *A*_8_ is up to 0.9, and the repeatability between the two is relatively large. The correlation coefficient diagram of man-made vulnerability is shown in Figure 3b. Among them, the correlation coefficient between *B*_5_ and *B*_7_ is 0.8, which is the strongest. In addition, the correlations between indicator *B*_3_ and other indicators are relatively large. The absolute value of the correlation coefficient between *B*_3_ and *B*_5_, *B*_3_ and *B*_6_ are all greater than 0.7. The correlations between other indicators are not so strong. The correlation coefficient diagram of vulnerability of carrying capacity is shown in Figure 3c. Compared with the indicators in the first two aspects, the redundancy between the indicators in the aspect of the vulnerability of carrying capacity is significantly greater. Among them, the absolute values of the correlation coefficient between *C*_2_ and other indicators are all not less than 0.3. In summary, there are more or less correlations among various indicators. At the same time, the definition and connotation of each specific indicator and the actual situation of the evaluation industry explain that there is duplication of information in the indicators initially constructed in the article. This information may have an impact on the final prediction of water resources vulnerability of the river basin. Therefore, it is very important to choose a suitable method to eliminate the impact of the constructed index as much as possible. On the basis of the indicator structure and classification ability unchanged, this article uses the neighborhood rough set theory to reduce the attributes of the remaining indicators for the important indicators selected by the qualitative analysis, which is more conducive to the subsequent assessment and prediction work.

#### 3.1.2. Determination of Decision-Making Attributes

When we use the neighborhood rough set to reduce the attributes of an assessment indicator system, the collected raw data does not need to be discretized, and can be directly used as conditional attributes. Due to the lack of decision-making attributes, we need to use linear weighted method to determine the comprehensive evaluation value of natural vulnerability, man-made vulnerability and vulnerability of carrying capacity. And then, the discretized comprehensive evaluation values are used as decision attributes to construct a neighborhood rough set decision system NDS. The discretization process is to transform the original continuous data into several “segments”. In this way, continuous data is converted into discrete data to reduce the complexity of continuous data and reduce the influence of extreme values on the data structure. The commonly used discretization methods currently used in rough set methods include equal frequency division discrete method, equal distance division discrete method, self-organizing neural network method, discrete method based on information entropy, etc. This paper uses k-means clustering discretization method for discretization, which is an unsupervised discretization method. It is carried out by the k-means clustering method, which can overcome the shortcomings of the isometric method and the equal frequency method that do not consider the data characteristics and related attributes of the index to a certain extent. The calculation is relatively simple, and it is also suitable for the actual situation with a small amount of sample data, so it is more suitable for this paper.

Among them, this paper uses the game weighting method [74] to determine the constructed water resource vulnerability indicator weights and uses the calculated weights to calculate the comprehensive evaluation value of natural vulnerability, man-made vulnerability and vulnerability of carrying capacity. The calculation steps are as follows:

*Step 1*: Perform non-dimensional standardization on the original data. The non-dimensional standardization processing formula of the positive indicator is as follows:
  (4) $$X_{ij}=\frac{x_{ij}-minx_{ij}}{maxx_{ij}-minx_{ij}}$$

The non-dimensional standardization processing formula of the negative indicator is as follows:

(5) $$X_{ij}=\frac{maxx_{ij}-x_{ij}}{maxx_{ij}-minx_{ij}}$$

In the formula, $x_{ij}$ represents the original value of the *j*-th indicator in the *i*-th year; $maxx_{ij}$ represents the maximum value of the *j*-th indicator; $minx_{ij}$ represents the minimum value of the *j*-th indicator.

*Step 2*: Determine the weight of the entropy method [75]. First of all, we calculate the information entropy $E_{j}$ of the *j*-th index:
  (6) $$E_{j}=-\frac{1}{\ln\left(n\right)}\overset{j}{\underset{i=1}{\sum }}(f_{ij}lnf_{ij})$$

In this formula, $f_{ij}=X_{ij}/\sum_{i=1}^{j}X_{ij}$; when $f_{ij}=0$, $f_{ij}lnf_{ij}=0$. Then, the entropy weight of the *j*-th index $w1_{j}$ is:

(7) $$w1_{j}=\frac{1-E_{j}}{\sum_{j=1}^{n}\left(1-E_{j}\right)}=\frac{D_{j}}{\sum_{j=1}^{n}\left(D_{j}\right)}$$

In this formula, $D_{j}$ is the indicator difference degree, and $D_{j}=1-E_{j}$.

*Step 3*: Determine the weight of the CRITIC (criteria importance though intercriteria correlation) law [76]. First of all, we quantitatively calculate the information amount $C_{i}$ of the assessment indicator:
  (8) $$C_{i}=S_{j}*R_{j}=\sqrt{\frac{\sum_{i=1}^{n}(X_{ij}-\frac{1}{n}\sum_{i=1}^{n}X_{ij})}{n-1}}*\overset{n}{\underset{t=1}{\sum }}\left(1-r_{rj}\right)$$

Here, $r_{rj}$ represents the correlation coefficient between the assessment indicator *i* and *j*; $S_{j}$ is the contrast strength between the *j*-th indicator and other indicators, that is, the standard deviation of the *j*-th indicator; $R_{j}=\sum_{t=1}^{n}\left(1-r_{rj}\right)$ is the conflicting quantitative indicator between the *j*-th indicator and other indicators. Then, the weight of the CRITIC law of the *j*-th index $w2_{j}$ is:

(9) $$w2_{j}=\frac{C_{j}}{\sum_{j=1}^{m}C_{i}}$$

*Step 4*: Determine the comprehensive weight of the game theory method. This paper uses the game theory method to integrate the entropy method and the CRITIC method to comprehensively determine the weight of the indicator. At the same time, the relevance, dispersion and relative strength of the indicator data information have also been fully tested. Thereby, the weighting result tends to be balanced, and the scientificity of the indicator weight is improved. The steps for determining the comprehensive weight of the game theory method are as follows:

Firstly, the entropy method and CRITIC method are used to weight the indicators respectively, and a basic weight vector set $w_{k}=\left\{w_{k1},w_{k2}\right\}$ is constructed. The arbitrary linear combination between the above 2 different vectors is:

(10) $$w=\overset{2}{\underset{k=1}{\sum }}\alpha_{k}w_{k}^{T}\;(\alpha_{k}>0,\overset{2}{\underset{k=1}{\sum }}\alpha_{k}=1)$$

where w is a possible weight vector in the basic weight vector set; $\alpha_{k}$ is the linear combination coefficient. Then, we use game theory to optimize the 2 linear combination coefficients $\alpha_{k}$, so that the deviation between w and each $w_{km}$ is the smallest, namely:

(11) $$min\|\overset{2}{\underset{j=1}{\sum }}\alpha_{j}w_{j}^{T}-w_{i}{\|}_{2}\left(i=1,2\right)$$

The optimal first derivative condition of the above formula can be converted into the following formula:

(12) $$\left[\begin{array}{cc} w_{1}w_{1}^{T} & w_{1}w_{2}^{T}\\ w_{2}w_{1}^{T} & w_{2}w_{2}^{T} \end{array}\right]\left[\begin{array}{c} \alpha_{1}\\ \alpha_{2} \end{array}\right]=\left[\begin{array}{c} w_{1}w_{1}^{T}\\ w_{2}w_{2}^{T} \end{array}\right]$$

Finally, according to the above process, $\left(\alpha_{1},\alpha_{2}\right)$ is obtained and then normalized, that is, the objective weight $w_{j}$ of the game of the *j*-th index is calculated:

(13) $$w_{j}=\frac{\left|\alpha_{k}\right|}{\sum_{k=1}^{2}\left|\alpha_{k}\right|}$$

*Step 5*: Use the standardized values of related indicators and the weights of each indicator in the water resources vulnerability assessment indicator system to calculate the final comprehensive evaluation value of vulnerability in the three aspects of natural vulnerability, man-made vulnerability and vulnerability of carrying capacity. The calculation formula is as follows:
  (14) $$Z_{i}=\overset{n}{\underset{j=1}{\sum }}\left(w_{j}X_{ij}\right)$$

#### 3.1.3. Attribute Reduction of Indicators

Through the abovementioned forward greedy algorithm, the original data is reduced by attributes. After many parameter adjustments, the calculated 3-dimensional set is the smallest reduction of the original decision table, that is, the reduction set of natural vulnerability $A=\left\{A_{3},A_{4},A_{7}\right\}$, the reduction set of man-made vulnerability $B=\left\{B_{2},B_{6},B_{7},B_{8}\right\}$, the reduction set of vulnerability of carrying capacity $C=\left\{C_{1},C_{3}\right\}$. After inspection, they all meet the definition requirements of the above reduction.

The result calculated by the forward greedy algorithm satisfies Definition 5. The above minimum reduction set represents the processing result of the collected indicator data from the perspective of data mining of the neighborhood rough set, but it cannot fully consider the actual situation of the area, and some indicators that are important for the assessment of water resource vulnerability may be deleted by mistake. This article’s assessment of the water resources vulnerability of the river basin not only includes the overall status of the river basin, but also includes the three aspects of natural vulnerability, man-made vulnerability and vulnerability of carrying capacity. In order to facilitate the assessment of the vulnerability of water resources in the three aspects, *A*_1_ is added to the set *A*, and *C*_5_, *C*_6_ are added to the set *C*. Finally, a reduced indicator system is formed, as shown in Table 3 below, which serves as the basis for the random forest regression model. In addition, according to the correlation analysis of evaluation indicators in the Section 3.1.1, the absolute values of the correlation coefficient between indicators are controlled below 0.6 after the reduction, that is, the redundancy of the indicator system is further reduced.

### 3.2. Construction of Water Resources Vulnerability Assessment Model

#### 3.2.1. Interpolation of Regression Samples

We take the standard values of the indicator level threshold of the assessment indicator system after dimensionality reduction as the input vector and the vulnerability level values as the output vector to construct a random forest model. Among them, the average value of the thresholds on both sides of the level range is used as the standard value of each indicator. In this way, the standard value matrix of each indicator corresponding to seven levels is calculated, which has dimensions of 7 rows (levels) × 12 attributes. Then, we use the standard value matrix as the input data for the random forest regression model calculation. Furthermore, a vector composed of seven vulnerability level values is used as the output vector.

However, considering that the size of standard value matrix is small, it is likely to have an adverse effect on the accuracy of the regression. Therefore, it is necessary to eliminate this effect and expand the matrix size. Then, we use the two-dimensional cubic interpolation method to complete by the function *interp2* of the MATLAB software. Finally, the standard value matrix which has dimensions of 7 rows (levels) × 12 attributes is expanded to a matrix whose dimensions are 100 rows (levels) × 12 attributes. Also, the output vector is expanded to a 100-dimensional vector. And then, the interpolated matrix is used as the input data and the interpolated vector is used as output data of the random forest model regression model.

#### 3.2.2. Construction of Random Forest Regression

**Figure 4:**
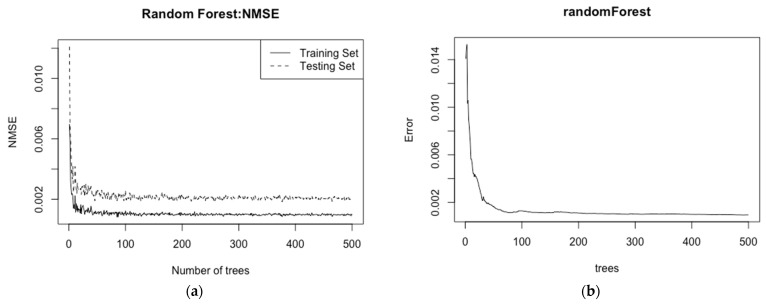
Random forest model training image: (**a**) relationship graph between model NMSE and *ntree*; (**b**) relationship graph between model error and *ntree*.

This paper uses the random forest (randomForest package) in the R language software for sample training. In order to better determine the tree and other parameters of the random forest, the existing sample set is randomly divided into two categories. The first category is used as the training set, and the second category is used as the test set. Then, we test the accuracy of a random forest with *n* trees, where *n* takes all integers from 0 to 500. It can be seen from Figure 4a that the higher the number of trees is, the smaller the error between the test set and the training set is. Therefore, the number of *ntree* in the random forest is set to 500. Figure 4b shows the relationship between the number of trees trained in the random forest and the error outside the bag. It can be seen that the higher the number of *ntree* is, the smaller the error of the model is and the more stable it becomes.

For the number of *mtry*, the traversal method is adopted. By setting the number of *mtry* in the interval [2,8], the NMSE of the training set and the test set is obtained as the *mtry* selection criterion. The calculation results are shown in Table 4. It can be seen that the accuracy of the random forest model is the best when the number of *mtry* reaches 4. Therefore, the number of parameters *mtry* is set to 4, and the random forest model is trained by the parameters set above.

In order to verify whether the random forest regression model is better than other models in the accuracy of the research in this article, we will compare the neural network model (net package), decision tree (rpart package) and support vector machine regression model (e1071 package) with excellent nonlinear regression functions. After setting and debugging the appropriate parameters of each model, the training of the model is carried out.

For the test of the trained model, this paper uses the 10-fold cross-validation method to judge the reliability of the result. We will use 10 randomly selected samples as 10 training sets, and take turns using nine of them as training data and one as test data for testing, so that each sample can be used as a test set. Finally, the average value of the mean square error (*MSE*), normalized mean square error (*NMSE*) and coefficient of determination (*R-squared* or *R^2^*) of all test results is obtained. The related error test formula is as follows [77]:

(15) $$MSE=\frac{1}{n}\sum^{\;}{\left(\overset{\wedge }{y_{i}}-y_{i}\right)}^{2}$$

(16) $$NMSE=\frac{\sum^{\;}{\left(y_{i}-\overset{\wedge }{y_{i}}\right)}^{2}}{\sum^{\;}{\left(y_{i}-\bar{y_{i}}\right)}^{2}}$$

(17) $$R^{2}=1-\frac{\sum_{i}{\left(\overset{\wedge }{y_{i}}-y_{i}\right)}^{2}}{\sum_{i}{\left(\overset{\wedge }{y_{i}}-\bar{y_{i}}\right)}^{2}}$$

Results of each error test obtained by the above method are shown in Table 5 above. It can be seen from the table that the selected regression models have good training accuracy, and the accuracy of random forest regression is higher than that of neural network models, support vector machine and decision trees. Therefore, this paper uses the random forest regression model as the water resource vulnerability assessment and prediction model of the Song-Liao River Basin.

### 3.3. Assessment of Water Resources Vulnerability

#### 3.3.1. Calculation of Current Situation of Water Resources Vulnerability

We use all indicator data on the natural vulnerability of the Song-Liao River Basin from 2000 to 2017 as input data. The random forest model trained by the above software package is used to obtain the comprehensive evaluation value of natural vulnerability in the past few years. The same operation is applied to the calculation of the vulnerability value of man-made vulnerability and vulnerability of carrying capacity. Then, we use the original indicator data of the Song-Liao River Basin from 2000 to 2017 as the input data and use the random forest model trained by the above software package to obtain the comprehensive evaluation value of the water resource vulnerability, as well as comprehensive evaluation value of natural vulnerability, man-made vulnerability and vulnerability of carrying capacity in the past few years. Then we rounded the relevant vulnerability data to get the relevant grade value. The calculation results are shown in Table 6 below.

#### 3.3.2. Current Situation Evaluation of River Basin’s Water Resources Vulnerability

**Figure 5:**
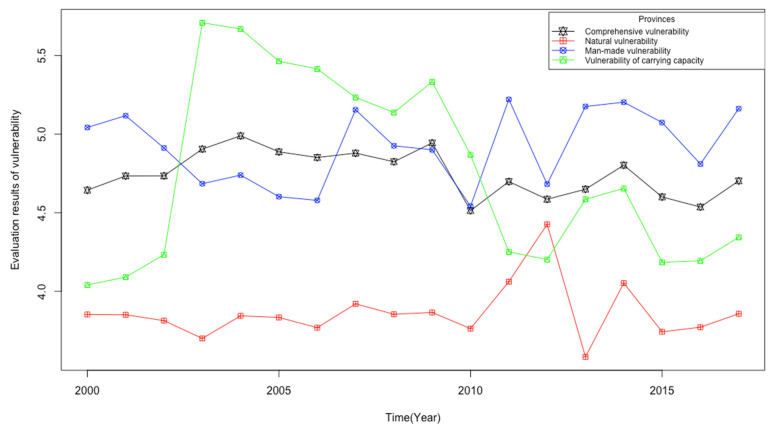
Evaluation results of water resources vulnerability in Song-Liao River Basin.

As can be seen from Figure 5 below, the Song-Liao River Basin is relatively fragile as a whole, basically in the level of moderate to high vulnerability. From a numerical point of view, the water resources vulnerability of the river basin has experienced a trend of first deterioration and then improvement from 2000 to 2017. However, its vulnerability level has been maintained at the level V, which shows that the overall water vulnerability of the river basin is still at a relatively poor level.

In terms of natural vulnerability, the water resource vulnerability of the Song-Liao River Basin is more serious, but the vulnerability is better compared with other aspects of the basin. According to relevant data, the water production modulus of the Song-Liao River Basin has been at a relatively poor level since 2000, with an average value of only 14.36 m^3^/km^2^, and some years have large fluctuations. There is a big gap between the Song-Liao River Basin and other water-producing areas, such as the Huai River Basin whose average water production modulus is 320,000 m^3^/km^2^. In the Song-Liao River Basin, basin itself has a vast area, the total amount of water resources is relatively abundant and there is a large gap between regions, which is an important factor that is likely to cause the uncertainty of water resources’ natural vulnerability. At the same time, in some areas, precipitation fluctuates greatly in some years, causing the basin to be affected by external natural climatic conditions. In addition, the imbalance of precipitation in the basin, especially the water shortage in inland areas, cannot be ignored, which will objectively aggravate the natural vulnerability of the Song-Liao basin. Additionally, in terms of water quality, the situation of regional water resources pollution has improved recently, but the extent is not very large. From the water quality qualified rate of the river basin, the relevant rate of Song-Liao River Basin in 2017 has increased by 2.96% compared with the previous year. Therefore, there is room for improvement in the future.

In terms of man-made vulnerability, the vulnerability of water resources in the Song-Liao River Basin is extremely high. From 2000 to 2017, the grade of water vulnerability basically stagnated to the level V of moderate to high vulnerability. Due to the lack of water resources in parts of the Song-Liao River Basin, there has been overexploitation in water resources exploitation, especially groundwater. The average proportion of groundwater resources being utilized from 2011 to 2017 was 48.49%, and it reached 51.98% in some years, which is a problem that cannot be ignored. The ratio of effectively irrigated farmland area is also steadily increasing in view of the transformation from extensive water use to intensive water use. In terms of environmental pollution emissions, the Song-Liao River Basin has a trend of improvement compared with the previous year. According to the “China Environmental Statistics Yearbook”, we can see that the main indicators of industrial pollution include per capita COD emissions and per capita ammonia and nitrogen emissions are gradually decreasing. In particular, COD emissions per capita in 2017 were reduced by about 32% compared to 2000, the initial year of the evaluation. In terms of water conservancy project construction, although there is a large investment every year, the effect is not very obvious. It can be seen from the three aspects of water conservancy regulation and storage capacity, water and soil erosion control, and embankment protection population construction that the relevant indicators are basically the same as those of the original year, which shows that the man-made vulnerability of water resources in the Song-Liao River Basin is difficult to manage, and the governance effect is not obvious. What’s more, the discharge of agricultural and domestic sewage is also a factor that cannot be ignored in the Song-Liao River Basin in terms of water quality. Also, this is an aspect needed to be paid attention to in the future to reduce the water vulnerability of the river basin.

In terms of vulnerability of carrying capacity, the overall water resource vulnerability of the Song-Liao River Basin has deteriorated first and then improved, and it is developing in a better direction. At the same time, the vulnerability of carrying capacity has improved the most compared to other vulnerabilities, whose grade has been changed from the past level V of moderate to high vulnerability to the level IV of moderate vulnerability. As the population in the Song-Liao River Basin has grown at a steady rate each year in recent years, the carrying pressure in the basin has also increased. On the other hand, with the advancement of active and effective measures in the basin, the pressure on the Song-Liao basin in other fields is also decreasing. Regarding the pressure on the amount of water resources, the ratio of the supply of groundwater resources to the total water supply is slowly decreasing, and it is expected to decrease to 45% in the next few years with the current trend. Due to the uncertainty of annual precipitation, the water carrying pressure in each year is also fluctuating. But from the trend of the population per 10,000 cubic meters of water, it is likely to further decline in the future. Therefore, there is much room for improvement in the vulnerability of river basin water resources.

### 3.4. Scenario Prediction and Analysis of River Basin’s Water Resources Vulnerability

#### 3.4.1. Raw Data under Different Scenarios in the Future

Setting different scenarios according to different levels of goals can more effectively control resources in advance, so as to prepare for advance control measures [78]. We set three scenarios—Scenario 1, Scenario 2, and Scenario 3—in the years of 2025 and 2030, respectively. At the same time, we collect original data under the three future scenarios and predict the future water vulnerability state of Song-Liao River Basin under these three scenarios. Recently, affected by human economic activities and lifestyles, forests have been continuously being cut down, cities have been expanding, and the stability of forest ecosystems have been declining [79]. This has further led to man-made greenhouse gas emissions reach the highest in current history, which has affected humanity and climate change in natural systems. All of the above factors in turn increase the uncertainty of water security and the vulnerability of water resources [80]. Therefore, the scenario data in scenario predictions that are greatly affected by the climate are collected based on the RCP4.5, RCP6.0 and RCP8.5 concentration emission scenarios set by the fifth IPCC assessment report to predict the future river runoff, precipitation and other data of the Song-Liao River Basin. We use this as the input data for the random forest prediction model of Scenario 1, Scenario 2, and Scenario 3 in the future forecast years.

In order to obtain other scenario data related to economic and social development, such as water efficiency, water conservancy projects, water pollution control, we need to first set the water resources management goals corresponding with the scenario indicators. The target data are based on the “Opinions of the State Council on Implementing the Strictest Water Resources Management System”, “Comprehensive Planning of the Song-Liao River Basin” (2012–2030) and the forecast results of the future socioeconomic water of the Song-Liao River Basin. We assume that Scenario 1, Scenario 2, and Scenario 3 respectively follow the principles of pessimism, mediocrity, and optimism, and they complete the progress according to the goal set based on a certain proportion. Among them, Scenario 1 represents that the most stringent water resources management system can be implemented in the water resources management of the river basin, and it can basically reach or exceed the target value of the relevant plan; Scenario 2 represents a medium management scenario, which can make the target variable reach 2/3 of the target value; Scenario 3 represents a weak management situation, which can make the target variable reach only 1/3 of the target value. Then, we use these as scenario data under 3 different management efforts in the future.

#### 3.4.2. Forecast Result Analysis

The water resources vulnerability prediction results and related grades of the Song-Liao River Basin under different scenarios in the future can be obtained by inputting the raw data under the above 3 different scenarios into the trained random forest regression model, which is shown in Table 7 below.

The above prediction results show that the vulnerability of water resources in the future will be greatly improved compared with the previous evaluation years, and the vulnerability prediction results of the three scenarios in 2030 are better than those in 2025. Additionally, the water resources vulnerability situation under Scenario 1 is the best, and the water resource vulnerability grade will reach the level III is the level of moderate to low vulnerability in 2030. Also, Scenario 2 is only in Scenario 1, and the prediction result of water vulnerability under Scenario 3 is the worst. This shows that if the Song-Liao River Basin can implement water resources management and control measures in accordance with relevant water resources management systems in the future. Scenario 1 can effectively change the current deterioration situation of the water resources vulnerability in the Song-Liao River Basin, while water resources vulnerability in Scenarios 2 and 3 increases slowly. The governance effect of Scenario 2 and Scenario 3 in 2030 is not as good as the governance effect of Scenario 1 in 2025, and the vulnerability of water resources in the basin will further deteriorate.

In terms of natural vulnerability, the natural vulnerability under scenario 1 will be raised to the level III of moderate to low vulnerability, which has a trend of further improvement compared to previous years. In terms of man-made vulnerability, improvement under the three different scenarios is relatively slow. In 2025, the vulnerability of the three scenarios will be level IV and V. It will be stabilized in a level IV of moderate vulnerability until 2030. This will also become one of the difficulties in managing the vulnerability of water resources in the Song-Liao River Basin in the future. In terms of vulnerability of carrying capacity, the water resource vulnerability state of Scenario 1 will be significantly faster than the state of Scenario 2 and Scenario 3. It can be seen from the above that if favorable measures can be taken, such as the construction of water conservancy projects, it can effectively reduce the risk of future disasters and further enhance the security of water resources in the river basin.

## 4. Conclusions

This paper uses the forward greedy algorithm of the neighborhood rough set method to reduce the dimensionality of the original indicator system to remove redundant attributes, which is based on the constructed indicator system of water resources vulnerability assessment in Song-Liao River Basin. Then, assessment indicators’ standard values after dimensionality reduction are taken as the evaluation sample, and the random forest regression (abbreviated as RF) model is used to assess the water resources vulnerability of the river basin. Finally, based on data under three different climate and socio-economic scenarios in the future, scenario predictions are made on the vulnerability of future water resources.

We apply the above methods to carry out an empirical analysis of the Song-Liao River Basin and obtain the current situation and prediction results of the water resources vulnerability assessment. First of all, from the perspective of the current assessment, the water resource vulnerability of the Song-Liao River Basin is relatively poor, and it is currently at the level V of moderate to high vulnerability. In particular, natural vulnerability and man-made vulnerability improve relatively slowly, which are at the level IV of moderate vulnerability and the level V of moderate to high vulnerability, respectively. The vulnerability of carrying capacity improves relatively more obviously, and it has improved from the level V of moderate to high vulnerability to the level IV of moderate vulnerability. Secondly, judging from the forecast results, the vulnerability of water resources in Scenario 1 will be significantly improved in the future. However, the progress of water vulnerability in Scenario 2 and Scenario 3 is very slow and may even deteriorate. It can be seen that whether the most stringent water resources management system and related regulations can be well implemented in the future is an important factor in determining whether the vulnerability of water resources can be improved.

This article has certain innovations and characteristics in indicator selection, assessment model and scenario prediction. First, in terms of indicator selection, some research indicators are not well-targeted, and the influence of correlation between the indicators and information redundancy on the accuracy of the model are not well considered, which will cause interference with the accuracy of the assessment results. On the basis of the indicator structure and classification ability unchanged, this article uses the neighborhood rough set theory to reduce the attributes of the remaining indicators, which is more conducive to the subsequent assessment and prediction work. In addition, compared with the traditional rough set, the neighborhood rough set does not depend on the discretized data, and there is no need to use the discretization method to divide the data. Moreover, as a basis for the training of the random forest regression model, it can objectively improve the efficiency of the random forest algorithm and the speed of model establishment. Second, in the assessment model, this paper compares multiple regression models with excellent nonlinear regression functions. We train the random forest, neural network model, decision tree and support vector machine regression model after setting and debugging the parameters properly. After judging the reliability of the results by using the 10-fold cross-validation method, it is found that the accuracy of the random forest regression model is higher than other models in this paper. Therefore, this paper selects the random forest regression model to assess the water vulnerability of Song-Liao River Basin. Third, the current prediction-related research is relatively small, which has caused the adjustment and control not to be prepared in time for early. Therefore, the future prediction of the vulnerability of water resources in the Song-Liao River Basin is worthy of attention. We refer to some important documents and set up three different scenarios in accordance with the principles of pessimism, mediocrity and optimism. Then, we input the raw data under three different scenarios into the random forest regression model with better training effect to study the vulnerability of water resources.

Restricted by data search, the assessment and prediction of the water vulnerability of the Song-Liao River Basin is limited to the basin’s overall situation in a certain year. In the internal system of the Song-Liao River Basin, due to the different geography, climate, economic and social conditions of each internal zone, the assessment indicators applicable to each internal system will be different. In future studies, we will focus on exploring the vulnerability of water resources at a smaller scale. Also, we will discuss the uncertainty of each sub-system of water resources and a dynamic adaptive forecasting management plan made in response to the predicted water vulnerability in the future. In addition, in view of the previous studies on the water-energy-food nexus [76,81], subsequent studies on water vulnerability should not only combine water resources with social, economic, and ecological external factors, but also should put water resources into the water-energy-food nexus to study the vulnerability of water-energy-food coupling.

## Figures and Tables

**Table 1:** Assessment indicator system of water resources vulnerability in Song-Liao River Basin.

First-Level	Second-Level	Number	Indicator	Attribute ^1^
Natural vulnerability	Water quantity	*A*_1_	Water production modulus	Negative
		*A*_2_	Variation coefficient of annual precipitation	Positive
		*A*_3_	Change rate of annual precipitation	Positive
	Water quality	*A*_4_	Water quality examination pass rate in water function area	Negative
		*A*_5_	Qualified ratio of water quality of river basin	Negative
		*A*_6_	Decline rate of water quality examination pass rate	Positive
	Disasters	*A*_7_	Proportion of area affected by flood and drought	Positive
		*A*_8_	Water production coefficient	Negative
Man-made vulnerability	Water quantity	*B*_1_	Proportion of surface water resources being utilized	Positive
		*B*_2_	Proportion of groundwater resources being utilized	Positive
	Water quality	*B*_3_	Total COD emission per 10,000 people	Positive
		*B*_4_	Total ammonia and nitrogen emission per 10,000 people	Positive
	Disasters	*B*_5_	Proportion of farmland area being the effectively irrigated	Negative
		*B*_6_	Proportion of population under levee protection	Negative
		*B*_7_	Proportion of soil erosion being controlled	Negative
		*B*_8_	Water conservancy project storage capacity	Negative
Vulnerability of carrying capacity	Water quantity	*C*_1_	Ratio of groundwater supply to total water supply	Positive
		*C*_2_	Per capita water consumption	Positive
		*C*_3_	Water consumption for irrigation per mu	Positive
	Water quality	*C*_4_	Population density	Positive
		*C*_5_	Wastewater generation per 10,000-yuan GDP	Positive
		*C*_6_	Ecosystem water consumption rate	Negative
	Disasters	*C*_7_	Population per 10,000 cubic meters of water	Positive
		*C*_8_	Reclamation index	Positive

^1^ The positive indicator indicates that the larger the value of the assessment index is, the higher the water resources vulnerability is; the negative indicator indicates that the smaller the value of the assessment index is, the lower the water resources vulnerability is.

**Table 2:** Grade standard for assessment of water resources vulnerability ^1^.

	Level I	Level II	Level III	Level IV	Level V	Level VI	Level VII
A1	(60, 100]	(50, 60]	(40, 50]	(30, 40]	(20, 30]	(10, 20]	(0, 10]
A2	(0, 0.1]	(0.1, 0.2]	(0.2, 0.3]	(0.3, 0.4]	(0.4, 0.5]	(0.5, 0.6]	(0.6, 0.8]
A3	(0, 0.1]	(0.1, 0.2]	(0.2, 0.3]	(0.3, 0.4]	(0.4, 0.5]	(0.5, 0.6]	(0.6, 0.7]
A4	(0.9, 1]	(0.8, 0.9]	(0.7, 0.8]	(0.6, 0.7]	(0.5, 0.6]	(0.4, 0.5]	(0.2, 0.4]
A5	(0.8, 1]	(0.7, 0.8]	(0.6, 0.7]	(0.5, 0.6]	(0.4, 0.5]	(0.25, 0.4]	(0.15, 0.25]
A6	(−0.8, 0]	(0, 0.05]	(0.05, 0.1]	(0.1, 0.2]	(0.2, 0.3]	(0.3, 0.4]	(0.4, 0.5]
A7	(0, 0.05]	(0.05, 0.1]	(0.1, 0.15]	(0.15, 0.2]	(0.2, 0.25]	(0.25, 0.3]	(0.3, 1]
A8	(0.7, 0.8]	(0.6, 0.7]	(0.5, 0.6]	(0.4, 0.5]	(0.3, 0.4]	(0.2, 0.3]	(0.1, 0.2]
B1	(0, 0.2]	(0.2, 0.25]	(0.25, 0.4]	(0.4, 0.55]	(0.55, 0.7]	(0.7, 0.85]	(0.85, 1]
B2	(0, 0.2]	(0.2, 0.25]	(0.25, 0.4]	(0.4, 0.55]	(0.55, 0.7]	(0.7, 0.85]	(0.85, 2]
B3	(15, 30]	(30, 45]	(45, 60]	(60, 75]	(75, 90]	(90, 105]	(105, 160]
B4	(4, 6]	(6, 8]	(8, 10]	(10, 12]	(12, 14]	(14, 16]	(16, 18]
B5	(0.95, 1]	(0.9, 0.95]	(0.85, 0.9]	(0.8, 0.85]	(0.75, 0.8]	(0.7, 0.75]	(0.65, 0.7]
B6	(0.9, 1]	(0.8, 0.9]	(0.7, 0.8]	(0.6, 0.7]	(0.5, 0.6]	(0.4, 0.5]	(0.2, 0.4]
B7	(0.9, 1]	(0.75, 0.9]	(0.6, 0.75]	(0.45, 0.6]	(0.3, 0.45]	(0.15, 0.3]	(0, 0.15]
B8	(0.9, 1.2]	(0.75, 0.9]	(0.6, 0.75]	(0.45, 0.6]	(0.3, 0.45]	(0.15, 0.3]	(0, 0.15]
C1	(0, 0.1]	(0.1, 0.2]	(0.2, 0.3]	(0.3, 0.4]	(0.4, 0.5]	(0.5, 0.6]	(0.6, 0.7]
C2	(180, 200]	(200, 300]	(300, 400]	(400, 450]	(450, 500]	(500, 550]	(550, 600]
C3	(180, 200]	(200, 300]	(300, 400]	(400, 450]	(450, 500]	(500, 550]	(550, 600]
C4	(0.008, 0.01]	(0.01, 0.02]	(0.02, 0.03]	(0.03, 0.04]	(0.04, 0.05]	(0.05, 0.06]	(0.06, 0.09]
C5	(2, 4]	(4, 6]	(6, 8]	(8, 10]	(10, 15]	(15, 25]	(25, 75]
C6	(0.06, 0.09]	(0.05, 0.06]	(0.04, 0.05]	(0.03, 0.04]	(0.02, 0.03]	(0.01, 0.02]	(0, 0.01]
C7	(0, 3.34]	(3.34, 5]	(5, 10]	(10, 15]	(15, 20]	(20, 30]	(30, 90]
C8	(0, 0.1]	(0.1, 0.2]	(0.2, 0.3]	(0.3, 0.4]	(0.4, 0.5]	(0.5, 0.6]	(0.6, 0.7]

^1^ The level I is the level of no vulnerability; The level II is the level of mild vulnerability; The level III is the level of moderate to low vulnerability; The level IV is the level of moderate vulnerability; The level V is the level of moderate to high vulnerability; The level VI is the level of highly vulnerability; The level VII is the level of extreme vulnerability.

**Table 3:** Indicator collection after reduction.

First-Level Indicator	Third Level Indicator
Natural vulnerability	Water production modulus *A*_1_
	Change rate of annual precipitation *A*_3_
	Water quality examination pass rate in water function area *A*_4_
	Proportion of area affected by flood and drought *A*_7_
Man-made vulnerability	Proportion of groundwater resources being utilized *B*_2_
	Proportion of population under levee protection *B*_6_
	Proportion of soil erosion being controlled *B*_7_
	Water conservancy project storage capacity *B*_8_
Vulnerability of carrying capacity	Ratio of groundwater supply to total water supply *C*_1_
	Water consumption for irrigation per mu *C*_3_
	Wastewater generation per 10,000-yuan GDP *C*_5_
	Ecosystem water consumption rate *C*_6_

**Table 4:** The accuracy of the test set and the training set under each different number of *mtry*.

Number of *mtry*	*NMSE* of Training Set	*NMSE* of Test Set
2	0.001004	0.005345
3	0.000937	0.005332
4	0.000896	0.005255
5	0.000977	0.005369
6	0.000927	0.005399
7	0.000927	0.005318
8	0.00096	0.00506

**Table 5:** Regression training accuracy of each model after attribute reduction.

Methods	*MSE*	*NMSE*	*R-Squared* ^1^
Random forest	0.0001529336	1.738314 × 10^−8^	0.9999968
Decision tree	0.00013895	0.01205421	0.9999924
Support vector machine	0.001653784	1.620705 × 10^−6^	0.9999991
Neural network	0.8765589	1.414752	0.9969101

^1^ The greater the R-squared is, the greater the precision of model is.

**Table 6:** Evaluation results of water resources vulnerability in the Song-Liao River Basin.

	Vulnerability Value (0–7)				Vulnerability Grade (I–VII)			
	Basin Vulnerability	Natural Vulnerability	Man-Made Vulnerability	Vulnerability of Carrying Capacity	Basin Vulnerability	Natural Vulnerability	Man-Made Vulnerability	Vulnerability of Carrying Capacity
2000	4.6439	3.8538	5.0428	4.0412	V	IV	V	IV
2001	4.7345	3.8520	5.1176	4.0910	V	IV	V	IV
2002	4.7350	3.8148	4.9121	4.2335	V	IV	V	IV
2003	4.9053	3.7021	4.6849	5.7085	V	IV	V	VI
2004	4.9896	3.8451	4.7396	5.6695	V	IV	V	VI
2005	4.8879	3.8350	4.6024	5.4638	V	IV	V	V
2006	4.8516	3.7702	4.5788	5.4150	V	IV	V	V
2007	4.8796	3.9211	5.1552	5.2339	V	IV	V	V
2008	4.8247	3.8551	4.9260	5.1380	V	IV	V	V
2009	4.9444	3.8665	4.9004	5.3324	V	IV	V	V
2010	4.5139	3.7642	4.5418	4.8695	V	IV	V	V
2011	4.6983	4.0618	5.2211	4.2512	V	IV	V	IV
2012	4.5862	4.4266	4.6821	4.2025	V	IV	V	IV
2013	4.6497	3.5852	5.1762	4.5860	V	IV	V	V
2014	4.8027	4.0528	5.2035	4.6551	V	IV	V	IV
2015	4.6008	3.7439	5.0740	4.1847	V	IV	V	IV
2016	4.5366	3.7731	4.8101	4.1949	V	IV	V	IV
2017	4.7030	3.8582	5.1625	4.3442	V	IV	V	IV

**Table 7:** Vulnerability value and grade of water resources in different scenarios in the future.

	Vulnerability Value (0–7)				Vulnerability Grade (I–VII)			
	Basin Vulnerability	Natural Vulnerability	Man-Made Vulnerability	Vulnerability of Carrying Capacity	Basin Vulnerability	Natural Vulnerability	Man-Made Vulnerability	Vulnerability of Carrying Capacity
2025 S1 ^1^	3.9930	2.6677	4.4839	4.1512	IV	III	IV	IV
2025 S2 ^1^	4.5857	3.6436	4.8763	4.6821	V	IV	V	V
2025 S3 ^1^	5.0388	3.8908	5.1832	5.4807	V	IV	V	V
2030 S1	3.0448	2.3793	3.5221	2.3116	III	III	IV	III
2030 S2	3.7156	3.3894	4.0384	3.4934	IV	IV	IV	III
2030 S3	4.3642	3.8460	4.2620	4.2809	IV	IV	IV	IV

^1^ S1, S2 and S3 are short for Scenario 1, Scenario 2 and Scenario 3.

## Data Availability

Not applicable.
